# Bedside Intestinal Ultrasound Performed in an Inflammatory Bowel Disease Urgent Assessment Clinic Improves Clinical Decision-Making and Resource Utilization

**DOI:** 10.1093/crocol/otad050

**Published:** 2023-09-21

**Authors:** Joëlle St-Pierre, Maxime Delisle, Hengameh Kheirkhahrahimabadi, Thomas M Goodsall, Robert V Bryant, Britt Christensen, Rose Vaughan, Aysha Al-Ani, Richard J M Ingram, Joan Heatherington, Dan Carter, Cathy Lu, Christopher Ma, Kerri L Novak

**Affiliations:** IBD Unit, Division of Gastroenterology and Hepatology, Department of Medicine, University of Calgary, Alberta, Canada; IBD Unit, Division of Gastroenterology and Hepatology, Department of Medicine, University of Calgary, Alberta, Canada; Division of Rheumatology, Department of Medicine, University of Calgary, Alberta, Canada; IBD Service, Department of Gastroenterology, The Queen Elizabeth Hospital, Adelaide, South Australia, Australia; School of Medicine, Faculty of Health Science, University of Adelaide, Adelaide, South Australia, Australia; IBD Service, Department of Gastroenterology, The Queen Elizabeth Hospital, Adelaide, South Australia, Australia; School of Medicine, Faculty of Health Science, University of Adelaide, Adelaide, South Australia, Australia; Department of Gastroenterology, The Royal Melbourne Hospital and University of Melbourne, Melbourne, Victoria, Australia; Department of Gastroenterology, The Royal Melbourne Hospital and University of Melbourne, Melbourne, Victoria, Australia; Department of Gastroenterology, The Royal Melbourne Hospital and University of Melbourne, Melbourne, Victoria, Australia; IBD Unit, Division of Gastroenterology and Hepatology, Department of Medicine, University of Calgary, Alberta, Canada; IBD Unit, Division of Gastroenterology and Hepatology, Department of Medicine, University of Calgary, Alberta, Canada; Department of Gastroenterology, Chaim Sheba Medical Center, Tel Hashomer, Israel and Sackler Faculty of Medicine, Tel Aviv University, Tel Aviv, Israel; IBD Unit, Division of Gastroenterology and Hepatology, Department of Medicine, University of Calgary, Alberta, Canada; IBD Unit, Division of Gastroenterology and Hepatology, Department of Medicine, University of Calgary, Alberta, Canada; Department of Community Health Sciences, University of Calgary, Alberta, Canada; IBD Unit, Division of Gastroenterology and Hepatology, Department of Medicine, University of Calgary, Alberta, Canada

**Keywords:** inflammatory bowel disease, Crohn’s disease, ulcerative colitis, point-of-care ultrasound, intestinal ultrasound, COVID-19

## Abstract

**Background:**

Patients with inflammatory bowel disease (IBD) require accessible, timely, and noninvasive strategies to monitor disease. The aim was to assess the integration of intestinal ultrasound (IUS) on decision-making and endoscopy utilization in a standardized care pathway.

**Methods:**

This prospective, multicenter, international, observational cohort study included patients seen within a centralized model for IBD care was conducted during the COVID pandemic. Patients were evaluated with IUS alone or in combination with an in-clinic, unsedated sigmoidoscopy. Demographic, clinical, laboratory, and imaging data, clinical decisions, and need for urgent endoscopy, hospitalization, and surgeries were recorded.

**Results:**

Of the 158 patients included, the majority had an established diagnosis of Crohn’s disease (*n* = 123, 78%), and 47% (*n* = 75) of patients were on biologic therapy. IUS identified active inflammation in 65% (*n* = 102) of patients, and strictures in 14% (*n* = 22). Fecal calprotectin levels correlated with inflammation detected on IUS (median of 50 μg/g [Q1–Q3: 26–107 μg/g] without inflammation and 270 μg/g [Q1–Q3: 61–556 μg/g] with inflammation; *p* = 0.0271). In the majority of patients, clinical assessment with IUS led to an acute change in IBD-specific medications (57%, *n* = 90) and avoided or delayed the need for urgent endoscopy (85%, *n* = 134). Four patients were referred for urgent surgical consultation.

**Conclusions:**

Point-of-care IUS used in a flare clinic pathway is a useful strategy to improve effective IBD care delivery and to assist in therapeutic management decisions, in many cases avoiding the acute need for endoscopy.

## Graphical Abstract

**Graphical Abstract:**
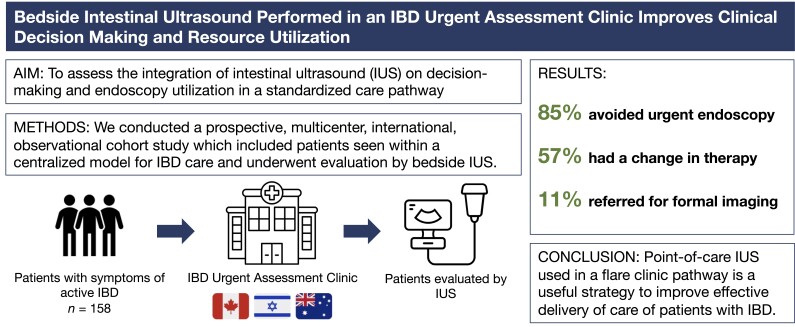

## Introduction

Inflammatory bowel diseases (IBDs), including Crohn’s disease (CD) and ulcerative colitis (UC), are common, immune-mediated disorders characterized by chronic inflammation and damage to the luminal gastrointestinal tract. Patients with IBD need frequent monitoring of their disease activity as well as dedicated, long-term follow-up, ideally in specialized clinics. They also require chronic immune suppression, and assessment of their response to treatment, to prevent complications and reduce disability associated with uncontrolled inflammation. Given the need for frequent encounters and assessments, there is a demand for the use of effective noninvasive techniques to assess active inflammation. Intestinal ultrasound (IUS) is an increasingly recognized innovative tool used at the bedside to accurately image the small and large bowel in cross-section, to detect disease activity, extent, and development of complications, and to monitor for postoperative recurrence.^1–5^ Mounting evidence supports accuracy compared to either gold-standard endoscopy,^6^ or to magnetic resonance enterography.^7,8^ IUS is patient-centered, well-tolerated, cost-effective, and easily repeatable, and can lead to clinical decision-making in up to 60% of cases when used in an expert clinic.^3,9^

The global spread of severe acute respiratory syndrome coronavirus 2 (SARS-CoV-2) has resulted in unprecedented risk to human life and led to overwhelming demands on health systems around the world. Health systems have been dramatically impacted, with substantial restrictions to in-person visits, transitions to online or virtual follow-up, limitations to routine blood and stool testing, and significant reductions to available endoscopy.^10,11^ Gastrointestinal societies and experts recommended offering only urgent procedures, deferring all nonurgent endoscopies.^12–17^ Recommendations to consider alternative disease assessment methods were made, highlighting the need for innovative approaches to ensure continued, high-quality IBD care for an often vulnerable, immunocompromised patient population. The COVID-19 pandemic has provided an opportunity to further demonstrate the utility of IUS, as the need for safe and noninvasive means to objectively evaluate patients with IBD and avoid endoscopy, where possible, is paramount.

In this multicenter, prospective observational cohort study, we summarize the experience of 4 expert centers in 3 countries (Canada, Israel, and Australia), all of which use integrated IUS in a centralized IBD clinical care pathway to improve the efficiency and quality of care for acutely flaring IBD patients during the COVID-19 pandemic.

## Methods

### Study Design and Patient Population

We conducted a prospective, multicenter observational cohort study at four centers including (1) the University of Calgary IBD Clinic, Calgary, Canada; (2) the Chaim Sheba Hospital, Tel Aviv, Israel; (3) The Queen Elizabeth Hospital, Adelaide, Australia; and (4) the Royal Melbourne Hospital, Melbourne, Australia. All centers are tertiary-level referral sites for IBD management, servicing large metropolitan areas and surrounding rural communities. At the start of the COVID-19 pandemic, a centralized model for urgent IBD care at all centers was collaboratively created to facilitate referrals for patients with established IBD with active symptoms and/or patients with findings or symptoms highly suspicious for a new diagnosis of IBD. The aim of the centralized model was to streamline care, optimize use of personal protective equipment, and provide consistent, safe, and timely access to care in the outpatient setting.

**Figure 1.**
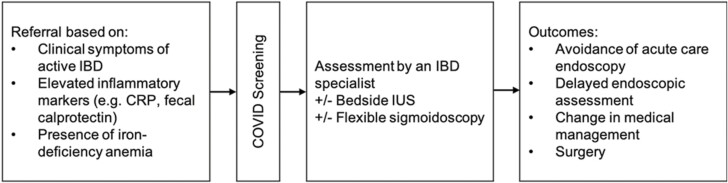
Care pathway to inflammatory bowel disease (IBD) specialist access during the COVID pandemic. Depicted above is the pathway from referral of patients to access to IBD specialty care and measured outcomes included in the study.

Eligible patients were aged 18 years and older and evaluated for suspicion of active IBD inflammation based on their referral to the urgent assessment clinic. Referrals to the 4 central care pathways were first screened based on worsening GI symptoms and/or worrisome laboratory investigations including elevated C-reactive protein (CRP), iron deficiency, and/or elevated fecal calprotectin (Figure 1). Second, patients were seen face to face in an outpatient clinic after being cleared for influenza-like illness symptoms, recent travel, and infectious exposures. In all 4 centers, symptoms limited to the gastrointestinal tract were deemed insufficient to trigger COVID-19 testing; therefore, no patients in this cohort were tested specifically for COVID-19 prior to the clinic visit in this early part of the pandemic. All patients were evaluated by an IBD-focused physician (KN and CL in Calgary, DC in Tel Aviv, TG and RB in Adelaide, and RV, AA or BC in Melbourne). Patients included in the study were seen from March 15th to June 30th, 2020. Finally, patients underwent noninvasive bedside IUS where appropriate, to inform therapeutic decision-making. Strict measures were employed to disinfect the ultrasound equipment. Where appropriate, patients were also evaluated by nonsedated in-clinic flexible sigmoidoscopy, where available.

### Intestinal Ultrasound

Transabdominal IUS was performed by experienced gastroenterologists with advanced training in IUS (K.L.N., C.L., T.M.G., R.V.B., D.C., B.C., A.A., and R.V.). IUS was performed using a multifrequency convex (3–10 MHz) transducer with a Samsung RS80A machine (Samsung Medison Co. Ltd, Seoul, Korea), 2–9 MHz transducer with a Philips Epiq 7 machine (Philips Healthcare, Bothell, WA), 18LX5 linear transducer with a Canon Aplio a550 (Canon Inc Operations, Tokyo, Japan), C1-5D broad-spectrum convex transducer and broad-spectrum 9L-D linear transducer (2–8 MHz) with a GE Logic S8 (General electrics, USA), 9C2 High-Frequency Curved Array Transducer (2–9 MHz), or 14L3 Linear Array Transducer (3–14 MHz) with a BK 3000 ultrasound machine (Bruel-Kjaer Medical Peabody, USA). All bowel segments were assessed in short and long axes. Active inflammation was defined as having a bowel wall thickness greater than 3 mm in any given segment, and hyperemia was seen on color Doppler imaging (standardized across sites to optimize low flow in the wall 4–7 cm/s) defined as modified Limberg 1 or above. A stricture was identified by the presence of all 3 of the following criteria: Presence of bowel wall thickening (more than 3 mm), presence of luminal narrowing <10 mm, and associated prestenotic dilation of more than 3 cm.^18^ Additional penetrating complications were documented, characterized by disruption of the usual bowel wall pattern, associated extraluminal areas, and variable hypoechogenicities with inflammation and/or air and fluid collections outside the lumen.

### Sigmoidoscopy

Where distal disease was known as rectal/left colon disease or highly suspected (eg, new-onset hematochezia or a patient with established UC), an unsedated flexible sigmoidoscopy was performed after standard, informed consent in the outpatient clinic setting. Biopsies were collected for histopathology.

### Data Analysis

Descriptive statistics were used to characterize the patient demographics and clinical features, in addition to IUS findings and outcomes. The proportion of patients achieving each of the following outcomes was summarized: Avoidance of urgent endoscopy, requirement for delayed nonurgent endoscopy, change in medical management (including requirement for corticosteroids, immunomodulators, and biologic or JAK inhibitor therapy), de-escalation of medical management, and surgery. As this was an observational cohort, no formal hypothesis testing was conducted. Other than CRP and fecal calprotectin levels, missing data represented less than 5% of all other variables collected.

The primary outcome measure was urgent hospital-based endoscopy avoidance (defined as avoidance of endoscopy performed within a planned hospitalization or as an urgent endoscopy within 2 weeks, as determined by the IBD specialist at the time of evaluation). Secondary outcomes included impact on clinical decisions, defined as any substantive changes in medical management (including the addition of corticosteroids, biologic dose escalation, dosing interval increase, drug, or class switch), and referral to colorectal surgery. Additional secondary outcomes included the proportion of patients with objective evidence of disease activity detected by IUS or both IUS and in-clinic sigmoidoscopy, proportion of patients presenting to the emergency department, and hospitalizations during the study period.

Continuous variables were described by their median value and interquartile (IQR) range. An independent-sample *t*-test was used to compare differences in fecal calprotectin levels between patients without inflammation on IUS in comparison to patients with active inflammation on IUS. Statistical analysis was performed with Stata (StataCorp, College Station, TX).

### Ethical Considerations

This study was approved and reviewed by the Conjoint Research Ethics Board at the University of Calgary, the Central Adelaide Local Health Network Human Research Ethics Board, and the local institutional ethics review boards of Melbourne Health, the Queen Elizabeth Hospital, and Chaim Sheba Medical Center. Each subject provided informed consent, which included the use of deidentified images for research and publication purposes.

## Results

A total of 158 patients aged 18 years or older from 4 centralized IBD flare outpatient clinics were included during the study period. Patient demographics and presenting details included in the initial referral are summarized in Table 1. Patient demographics, disease characteristics and behavior, medications at the time of referral, and median CRP value by recruitment site are included in Supplementary Table 1. The majority of the patients were female (*n* = 89, 56%), nonsmoking (*n* = 140, 89%), with a median age of 37 years (Q1–Q3: 26–49 years). Most patients evaluated at the IBD flare outpatient clinics had a diagnosis of CD (*n* = 123, 78%) and of these, most had ileal CD (*n* = 58, 47%) with inflammatory-type disease behavior (*n* = 61, 50%), and age of onset between 17 and 40 years (*n* = 92, 75%). Only 11% (*n* = 18) of patients seen had a diagnosis of UC, with the majority of those with left-sided colitis (*n* = 13, 72%). Seventeen patients (11% of patients assessed in clinic) were referred for symptoms suspicious for a new diagnosis of IBD and of these, 31% (4 of 17) represented a new diagnosis of IBD. CRP levels were available for 116 patients (73%). The median CRP of patients evaluated in our clinical care pathway was 5.1 mg/L (Q1–Q3: 1.8–13.0 mg/L).

**Table 1.** Patient demographics, diagnosis, medical therapy, and median CRP at the time of referral for urgent access to care.

Characteristics	Combined population *n* = 158 (100%)
Population characteristics	
Median age (years, [Q1–Q3])	37 [26–49]
Female (*n*, %)	89 (56)
Smoking status (*n*, %)	
Current smoker	18 (11)
Nonsmoker	140 (89)
Crohn’s disease (*n*, %)	123 (78)
Ulcerative colitis (*n*, % of total)	18 (11)
Symptoms without diagnosis (*n*, % of total)	17 (11)
New IBD diagnosis	4 (31)
Medications at assessment (*n*, % of total)	
No therapy	53 (34)
Monotherapy	71 (45)
Corticosteroid	4 (3)
5-ASA	12 (8)
Immunomodulator (IM)^a^	8 (5)
Anti-TNF^b^	29 (18)
Ustekinumab	9 (5)
Vedolizumab	7 (4)
Tofacitinib	0 (0)
Clinical trial medication^c^	1 (1)
Rectal therapy only	1 (1)
Multiple therapies	34 (22)
Biologic + IM	23 (14)
Corticosteroid + biologic/IM	6 (4)
Corticosteroid + 5-ASA	2 (1)
	1 (1)
5-ASA + IM	2 (1)
Median CRP (in mg/L)	
Total [Q1–Q3]	5.1 [1.8–13.0]
Crohn’s disease [Q1–Q3]	4.3 [1.7–12.2]
Ulcerative colitis [Q1–Q3]	8.0 [3.9–13.5]
	42 (27)

^a^Immunomodulators include thiopurine or methotrexate.

^b^Includes biosimilar CPT-13, originator infliximab, adalimumab, golimumab.

^c^Clinical trial drugs included rizankizumab and upacitanib.

Abbreviations: CRP = C-reactive protein; IM = immunomodulator; TNF = tumor necrosis factor.

The main symptoms experienced by the patient that prompted a referral to the IBD urgent assessment clinic included abdominal pain (*n* = 102, 65%), diarrhea (*n* = 88, 56%), rectal bleeding (*n* = 32, 20%), urgency (*n* = 31, 20%), obstructive symptoms (*n* = 24, 15%), and weight loss (*n* = 19, 12%). The majority (*n* = 75, 47%) were on monotherapy or on multiple therapies, whereas 34% (*n* = 53) were not on any treatment. The remainder of patients (*n* = 30, 19%) were on either rectal therapy alone, 5-ASA, immunomodulators, corticosteroids, or a combination of these (Table 1 and Supplementary Table 1).

All were evaluated with bedside IUS, with 18% (*n* = 30) evaluated with IUS in combination with in-clinic, unsedated sigmoidoscopy (Table 2 and Supplementary Table 2). Four patients from the combined cohort were pregnant, and all avoided further invasive or hospital-based testing. The quality of 2 IUS examinations was compromised because of body habitus, while 2 patients were not considered candidates for IUS due to obesity and complex pelvic anatomy, respectively.

**Table 2.** Disease activity as measured by ultrasound or both ultrasound and flexible sigmoidoscopy.

Disease activity measures	Combined population (*n* = 158)	
	US only, *n* = 128	US and SIG, *n* = 30
Active inflammation (*n*, %)	82 (64)	20 (67)
Median maximal BWT (mm, [Q1–Q3])	4.2 [2.8–5.8]	4.9 [3.0–7.2]
Stricture (*n*, %)	21 (16)	1 (3)

Abbreviations: BWT = bowel wall thickness; SIG sigmoidoscopy; US = ultrasound.

**Figure 2.**
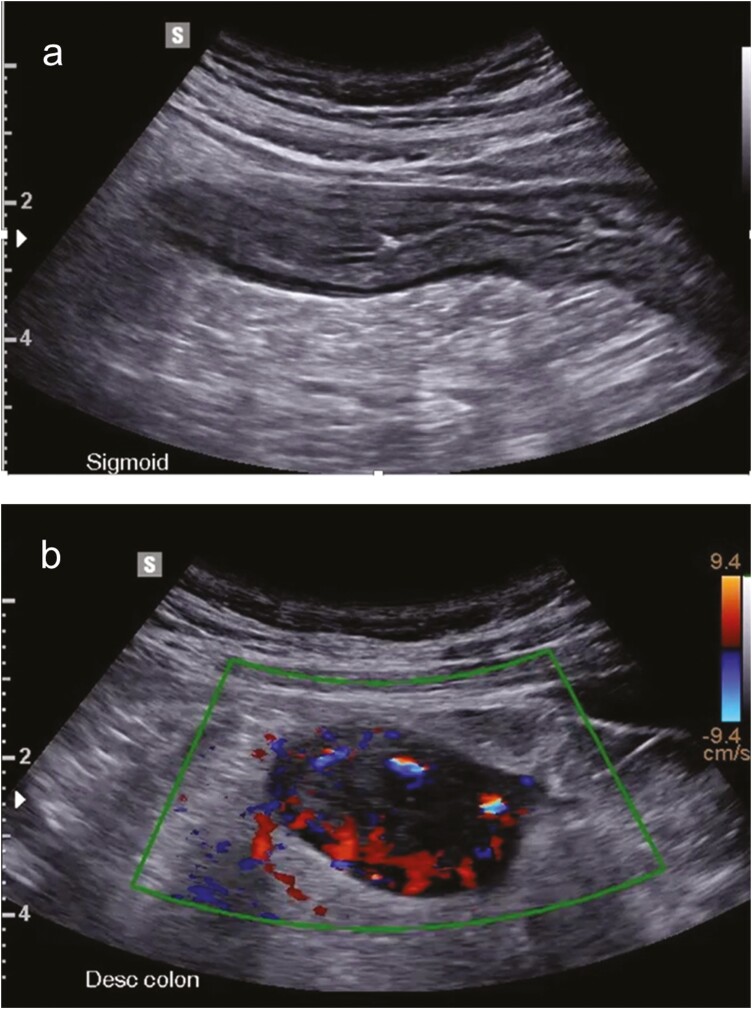
Colonic wall thickening in a patient with active Crohn’s disease (CD). A patient with known colonic CD was assessed at the inflammatory bowel disease (IBD) flare clinic for diarrhea and elevated C-reactive protein in the context of discontinuation of methotrexate due to fear of increased risk of COVID infection. (A) Longitudinal section of the sigmoid bowel with preserved echostratification, thickened wall up to 7 mm, and surrounding echogenic mesenteric fat. (B) Transverse image of the descending colon which is thickened, is hypoechoic, has echostratification loss and with abundant blood flow as seen on color Doppler signal. Images were acquired with a multifrequency convex transducer (3–10 MHz) with a Samsung RS80A machine (Samsung Medison Co. Ltd, Seoul, Korea).

**Figure 3.**
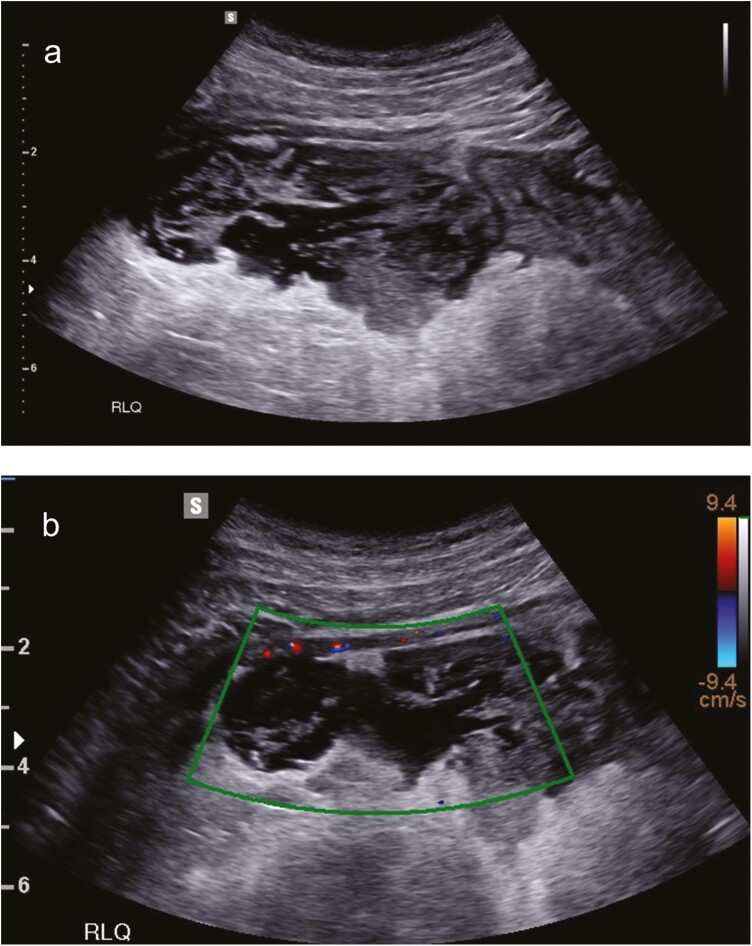
Patient with a postoperative anastomotic leak and abscess. A patient with Crohn’s disease (CD) and 9 days post-ileocecectomy was assessed at the inflammatory bowel disease (IBD) flare clinic for abdominal pain and leukocytosis. (A) A 8-cm abscess at the site of the anastomosis was identified, with surrounding hyperechoic inflammatory fat. (B) The abscess showed a lack of blood flow, as demonstrated by lack of Doppler signal, and consistent with pus. Images were acquired with a multifrequency convex transducer (3–10 MHz) with a Samsung RS80A machine (Samsung Medison Co. Ltd, Seoul, Korea).

Active inflammation was seen in 65% (*n* = 102) of all patients seen at the IBD flare outpatient clinics (82 of the 128 patients investigated by IUS only [64%] and 20 of the 30 patients investigated with both IUS and sigmoidoscopy [67%]) (Table 2 and inflammation demonstrated in Figure 2). The median maximal bowel thickness was 4.2 mm (Q1–Q3: 3.0–6.0 mm) (4.2 mm [Q1–Q3: 2.8–5.8 mm] for patients investigated by IUS only and 4.9 mm [Q1–Q3: 3.0–7.2 mm] for patients investigated by IUS and sigmoidoscopy), which is above the suggested normal BWT threshold of 3 mm for active inflammation.^19,20^ Flexible sigmoidoscopy and IUS were incongruent in 3 cases, and in all cases, active inflammation was limited to the rectal or perianal areas. Strictures were present in 22 patients (14%), all of whom had CD. Penetrating complications were rare but fistulas were seen in 3 patients and abscess in one patient (Figure 3). The latter was a patient with CD 9 days post-ileocecectomy and was found to have an 8-cm abscess at the anastomotic site. After IUS assessment, he was transferred to interventional radiology for a percutaneous drain and treated with oral antibiotics and avoided hospitalization.

**Figure 4.**
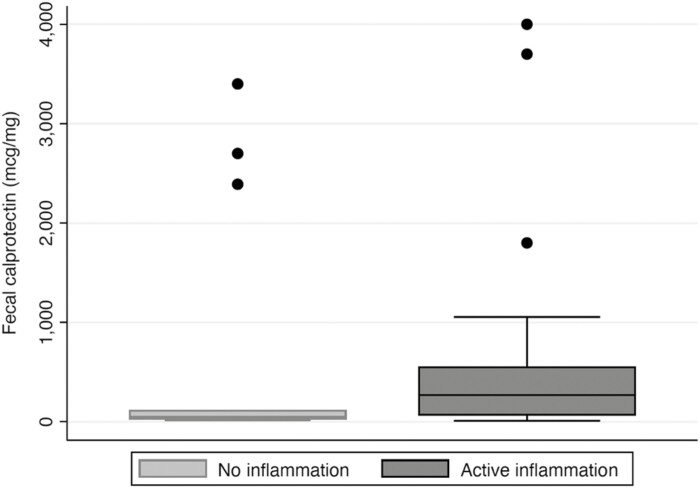
Fecal calprotectin levels are significantly lower in patients with no inflammation on intestinal ultrasound (IUS). Fecal calprotectin levels were available for 44 patients seen at the clinic, of which 29 showed active inflammation on IUS. Distribution of fecal calprotectin values is represented above. Differences in median fecal calprotectin levels between each group was statistically significant (*p* = .0271).

Fecal calprotectin was available for 44 patients undergoing IUS, of which 29 (66%) showed active inflammation on IUS. The median fecal calprotectin level for patients without inflammation on IUS was 50 μg/g (IQR 26–107 μg/g) as compared to 270 μg/g (IQR 61–556 μg/g), which was statistically significant (*p* = .0271) (Figure 4). Of the 10 patients with active disease on IUS and fecal calprotectin <100 μg/g, all but 1 had ileal inflammation in the context of CD, ranging from mild to moderate. One patient with UC had an incongruent fecal calprotectin level (18.5 μg/g) out of keeping with IUS findings showing pancolonic increased bowel wall thickness.

The majority (85%, *n* = 134) of patients evaluated at the IBD flare outpatient clinics avoided urgent endoscopy (86% of patients evaluated by IUS only; 80% of patients evaluated with both IUS and flexible sigmoidoscopy) (110 of the 128 patients investigated by IUS only [86%] and 24 of the 30 patients investigated with both IUS and sigmoidoscopy [80%]) (Table 3). All the patients sent for urgent endoscopy had a good quality IUS scan and the majority of these patients (*n* = 20, 83%) had inflammation on IUS. The reasons for referral for urgent colonoscopy included need to establish a diagnosis with biopsies (*n* = 3), endoscopic staging ahead of the initiation or change of biologic therapy (*n* = 5), endoscopy ahead of colorectal surgery (*n* = 2), diagnostic uncertainty (*n* = 3), and reasons not reported (*n* = 11). Twenty-one patients (13% of total cohort) were referred for nonurgent outpatient endoscopy, which includes colonoscopy (*n* = 16), flexible sigmoidoscopy (*n* = 2), and video capsule endoscopy (*n* = 3). None of the patients with impaired scan quality due to body habitus were referred for urgent or nonurgent endoscopy.

**Table 3.** Resource utilization after evaluation by ultrasound or both ultrasound and flexible sigmoidoscopy.

Resource utilization	Combined population (*n* = 158)	
	US only, *n* = 128	US and SIG, *n* = 30
Avoided urgent endoscopy (*n*, %)	110 (86)	24 (80)
Further outpatient investigations (*n*, %)	83 (65)	14 (47)
Fecal calprotectin	31 (24)	3 (10)
Other biochemical investigation^a^	21 (16)	5 (17)
Formal imaging^b^	16 (13)	2 (7)
Planned nonurgent endoscopy^c^ (*n*, %)	17 (13)	4 (13)

Abbreviations: SIG = sigmoidoscopy; US = ultrasound.

^a^Other biochemical investigation includes drug levels, pre-biological work-up, stool cultures, C diff testing.

^b^Formal imaging includes bowel US performed by a radiologist, MR enterography, or CT abdomen.

^c^Nonurgent endoscopy includes outpatient colonoscopy, flexible sigmoidoscopy, and video capsule endoscopy.

Nonurgent outpatient endoscopy, which includes colonoscopy, flexible sigmoidoscopy, and video capsule endoscopy, was recommended in 13% (*n* = 21) of patients assessed. None of the patients with impaired scan quality due to body habitus were referred for urgent or nonurgent endoscopy.

Further outpatient investigations, which included biochemical investigations (eg, fecal calprotectin, pre-biologic work-up, therapeutic drug levels) and further imaging studies (eg, MR enterography, CT enterography) were required in 61% (*n* = 97) of all patients assessed (83 of the 128 patients investigated by IUS only [65%] and 14 of the 30 patients investigated with both IUS and sigmoidoscopy [47%]). The majority of these (22%, *n* = 34) requests were for subsequent fecal calprotectin monitoring (31 of the 128 patients investigated by IUS only [24%] and 3 of the 30 patients investigated with both IUS and sigmoidoscopy [10%]). Formal imaging as performed by a radiologist was recommended in 11% (*n* = 18) of all patients seen at the clinic (16 of the 128 patients investigated by IUS only [13%] and 2 of the 30 patients investigated with both IUS and sigmoidoscopy [7%]). Of these, 10 MRI studies, 2 CT enterography, and 6 ultrasounds were ordered, with indications including sonographic assessment of perianal disease, assessment of small bowel inflammation by MRI or CT enterography, and confirmation of IUS findings.

Acute changes in IBD-specific medications were made in 57% (*n* = 90) of patients seen at the clinic, and most of these (*n* = 85, 66%) involved the addition of therapy or dose escalation (see Table 4). The majority (42%, *n* = 67) of IBD-specific therapeutic changes were the addition or dose escalation of biologic therapy (64 of the 128 patients investigated by IUS only [50%] and 3 of the 30 patients investigated with both IUS and sigmoidoscopy [10%]), followed by addition or optimization of immunomodulator therapy (*n* = 34 [22%] of total population; 31 of the 128 patients investigated by IUS only [24%] and 3 of the 30 patients investigated with both IUS and sigmoidoscopy [10%]). Corticosteroids were started or continued in 9% (*n* = 14) of patients (13 of the 128 patients investigated by IUS only [10%] and 1 of the 30 patients investigated with both IUS and sigmoidoscopy [3%]). JAK inhibitor start or optimization was only seen in patients who underwent both IUS and flexible sigmoidoscopy (*n* = 3, 10%). Other adjunct therapies such as the introduction of antidiarrheal medications, laxatives, tricyclic antidepressants, and antibiotics were added in 19% (*n* = 30) of patients assessed at the clinic. De-escalation of therapy was done in 5 patients (4%), 3 of which were done in the context of concerns for COVID infection while in remission, 1 for rapid taper of steroids started on the basis of symptoms and showing no objective evidence of inflammation and 1 for discontinuation of immunomodulator use based on remission on nonanti-TNF-alpha biologic therapy. Of note, 6 patients had more than 1 medication change, and data regarding medication changes were missing for 5 patients.

**Table 4.** Changes in medical management or surgical consultation after evaluation by ultrasound or both ultrasound and flexible sigmoidoscopy.

Outcome measures	Combined population (*n* = 158)	
	US only, *n* = 128	US and SIG, *n* = 30
Corticosteroid start/continued (*n*, %)	13 (10)	1 (3)
Immunosuppression start/optimization (*n*, %)	31 (24)	3 (10)
Biologic start/optimization (*n*, %)	64 (50)	3 (10)
JAK inhibitor start/optimization (*n*, %)	0 (0)	3 (10)
De-escalation of therapy (*n*, %)	5 (4)	0 (0)
Surgical consultation (*n*, %)	4 (3)	0 (0)

Four patients were referred for surgical consultation based on complications characterized by IUS. Of these, 2 patients required urgent hospital admission for ileocecal resection. One patient experienced ongoing obstructive symptoms from a severe, high-grade stricture, and the second patient was symptomatic from an ileocolonic fistula. Both complications were identified on bedside IUS.

## Discussion

Outpatient IBD urgent assessment clinics provide rapid access for patients with signs and symptoms of IBD disease relapse. The use of point-of-case IUS as part of a comprehensive assessment by an IBD specialist is increasingly used to assess active inflammation and guide management. In this multicenter, observational study, the integration of IUS in a clinical care pathway offered strategic advantages for IBD management both during times of limited endoscopy such as during the COVID-19 pandemic, with the purpose of wider implementation beyond the pandemic given clear advantages demonstrated. First, we showed avoidance of urgent hospital-based endoscopy in the majority of patients seen at the IBD flare clinics, despite active inflammation, using an accurate, safe noninvasive alternative. Second, the majority of patients had a substantive change in current medications such as biologic dose escalation, dosing interval increase, optimization, drug or class switch, and appropriate, expedited referral to colorectal surgery. Importantly, the appropriate timing of further investigations and/or treatment decisions was based on objective assessments by IUS, as opposed to traditionally dictated by reported clinical symptoms alone. This allowed deferral of urgent endoscopy and reduced exposure risks associated with both corticosteroids and invasive investigations, allowing for appropriate focus on therapies to treat symptoms of irritable bowel syndrome, a common overlapping entity with IBD.

The COVID-19 pandemic disrupted established management paradigms. The gold-standard endoscopic evaluation was heavily restricted in many centers, providing an opportunity to explore new and potentially beneficial ways of evaluating patients. The pandemic illustrated the capacity of IUS to inform clinical decision-making, obviating the need for invasive procedures in many instances. Considerate resource utilization was underlined during the acute phase of the pandemic, and continues to be of uttermost importance as healthcare systems continue to be overburdened by increased clinical demands. Despite most centers returning to regular workflows, the current study underlines the importance of IUS in the urgent evaluation of patients with symptoms of active disease while being mindful of hospital-based resources.

IUS is increasingly recognized as a noninvasive, radiation-free, low-cost tool to assess IBD activity and complications.^2^ The potential impact on real-time clinical decisions has been established in addition to response and healing as important potential in predicting patient outcome.^3–5,8^ This is particularly important for patients with CD for whom there is an early therapeutic window to change the disease course with appropriate treatment intervention.^21^ Conversely, patient symptoms are not always reflective of luminal inflammation: As many as 40% of patients with IBD will have concurrent irritable bowel syndrome.^22^ Avoiding unnecessary corticosteroids or therapeutic escalation of immunosuppression is crucial, especially with the increased risk of severe COVID infection reported with these medications in the SECURE-IBD registry.^23^ Our study showed a correlation between inflammation detected on IUS and fecal calprotectin levels. Interestingly, a portion of patients with active inflammation on IUS.

Our study strengthens the evidence to support IUS in real-time clinical decision-making and management of patients with IBD. It is the first study of its kind performed across multiple centers, and does so in the setting of an urgent IBD assessment clinic.

Despite its known advantages, several barriers have prevented IUS from being routinely implemented in many jurisdictions, especially in the United States. As a result, IUS remains underused.^24^ Performance and interpretation of IUS require specialized training and expertise. Furthermore, while IUS is typically performed by gastroenterologists at the bedside, when ultrasound is performed by radiology technologists and interpreted by radiologists, several layers of potential complexity with respect to reimbursement, availability, and potential healthcare exposures are added. The pandemic circumstances have provided a window of opportunity to demonstrate the patient-centered focus and safety of bedside IUS, in addition to the potential for avoiding invasive testing and optimizing objective disease monitoring. Our data may now be used to advocate for wider availability of this cost-effective procedure.

We also acknowledge some important limitations inherent in this observational study. First, all 4 centers use IUS regularly for their clinical care, representing a potential bias in favor of the incorporation and utility of IUS. Second, this was not a randomized intervention. There was no control population, as all the patients had access to the centralized IBD care model using IUS as a monitoring tool. Given the need for rapid change and adaption to the needs of a global pandemic, no blinding was possible between the performer of IUS and the expert making clinical decisions. Third, clinical outcomes were not evaluated in this study given the above limitations. However, given the potential beyond the COVID-19 pandemic, it is important to disseminate this innovative and safe approach which is patient centered and potentially cost-effective. Notwithstanding these limitations, our study also has some key strengths, including the inclusion of the same model from multiple, international expert centers, in addition to an adequate sample size illustrating the feasibility and practicality of this safe intervention.

Given the ongoing impact of COVID-19 on access to endoscopy in healthcare systems, bedside IUS may be considered to improve the quality of IBD care during this pandemic, providing important insights into both planning for future pandemic associated changes to health care and for care outside of COVID, given the potential that routine IUS use would have on the day-to-day care of IBD. Thus, the successful integration of IUS in clinical pathways will inform future optimal monitoring strategies for IBD care delivery post-pandemic.

## Conclusions

This study shows that point-of-care IUS is a useful noninvasive tool to improve care delivery to patients with IBD by assisting in therapeutic management decisions and avoiding urgent endoscopy.

## Supplementary Material

otad050_suppl_Supplementary_Tables_1-2

## Data Availability

The cumulative data underlying this article are available in the article and its online supplementary material. A more detailed breakdown of data for individual patients, without identifying information, will be shared on reasonable request to the corresponding author.
