# Supplementary material for: Bedside Intestinal Ultrasound Performed in an Inflammatory Bowel Disease Urgent Assessment Clinic Improves Clinical Decision-Making and Resource Utilization
Source: Crohns Colitis 360. 2023 Sep 21;5(4):otad050. doi: 10.1093/crocol/otad050 (PMC10558199; doi:10.1093/crocol/otad050)
Supplement: otad050_suppl_Supplementary_Tables_1-2 [file otad050_suppl_supplementary_tables_1-2.docx]

**Supplemental Table 1. Patient demographics, disease characteristics and behaviour, medications and median CRP at time of referral, per site.**

| **Characteristics** | **Combined Population**  ***n* = 158 (100%)** | **Calgary (Canada)**  ***n* = 63 (40%)** | **Melbourne and Adelaide (Australia)**  ***n* = 60 (38%)** | **Tel Aviv (Israel)**  ***n* = 35 (22%)** |
| --- | --- | --- | --- | --- |
| **Population characteristics** | | | | |
| **Median age** (years, [Q1-Q3]) | 37 [26 – 49] | 40 [27 – 56] | 37 [27 – 44] | 33 [25 – 49] |
| **Female** (*n*, %) | 89 (56) | 36 (57) | 32 (53) | 21 (60) |
| **Smoking status** (*n*, %) |  |  |  |  |
| Current smoker | 18 (11) | 7 (11) | 7 (12) | 4 (11) |
| Non-smoker | 140 (89) | 56 (89) | 53 (88) | 31 (89) |
| **Crohn’s Disease** (*n*, %) | 123 (78) | 46 (73) | 47 (78) | 30 (86) |
| **Age at diagnosis** (*n*, % of patients with Crohn's disease) | | | | |
| A1 (≤16 years) | 11 (9) | 2 (4) | 8 (17) | 1 (3) |
| A2 (17-40 years) | 92 (75) | 36 (78) | 32 (68) | 24 (80) |
| A3 (>40 years) | 15 (12) | 8 (17) | 3 (6) | 4 (13) |
| *Not reported* | 5 (4) | 0 (0) | 4 (9) | 1 (3) |
| **Disease Location** (*n*, % of patients with Crohn's disease) | | | | |
| L1 (ileal) | 58 (47) | 18 (39) | 19 (40) | 21 (70) |
| L2 (colonic) | 25 (20) | 17 (37) | 7 (15) | 1 (3) |
| L3 (ileocolonic) | 40 (32) | 11 (24) | 21 (45) | 8 (27) |
| **Disease Behaviour** (*n*, % of patients with Crohn's disease) | | | | |
| B1 (inflammatory) | 61 (50) | 20 (43) | 25 (42) | 16 (46) |
| B2 (stricturing) | 44 (36) | 17 (37) | 18 (30) | 9 (26) |
| B3 (penetrating) | 18 (15) | 10 (22) | 4 (7) | 4 (11) |
| *Phenotype not reported* | 1 (1) | 0 (0) | 0 (0) | 1 (3) |
| Perianal disease | 22 (18) | 7 (15) | 12 (20) | 3 (9) |
| *Perianal disease not reported* | 2 (2) | 0 (0) | 1 (2) | 1 (3) |
| **Ulcerative colitis** (*n*, % of total) | 18 (11) | 9 (14) | 9 (15) | 0 (0) |
| Location (n, % of patients with ulcerative colitis) | | | | |
| Pancolitis | 2 (11) | 2 (22) | 0 (0) | 0 (0) |
| Left sided colitis | 13 (72) | 5 (56) | 8 (88) | 0 (0) |
| Proctitis | 3 (17) | 2 (22) | 1 (11) | 0 (0) |
| **Symptoms without diagnosis** (*n*, % of total) | 17 (11) | 8 (13)) | 4 (7) | 5 (14) |
| New IBD diagnosis | 4 (31) | 3 (33) | 1 (25) | 0 (0) |
| **Medications at assessment** (*n*, % of total) | | | | |
| No Therapy | 53 (34) | 24 (38) | 13 (22) | 16 (46) |
| Monotherapy | 71 (45) | 31 (49) | 23 (38) | 17 (49) |
| Corticosteroid | 4 (3) | 2 (3) | 1 (2) | 1 (3) |
| 5-ASA | 12 (8) | 2 (3) | 7 (12) | 3 (9) |
| Immunomodulator (IM)* | 8 (5) | 3 (4) | 5 (8) | 0 (0) |
| Anti-TNF ^#^ | 29 (18) | 12 (19) | 7 (12) | 10 (29) |
| Ustekinumab | 9 (5) | 5 (7) | 3 (5) | 1 (3) |
| Vedolizumab | 7 (4) | 5 (7) | 0 (0) | 2 (6) |
| Tofacitinib | 0 (0) | 0 (0) | 0 (0) | 0 (0) |
| Clinical trial medication^+^ | 1 (1) | 1 (2) | 0 (0) | 0 (0) |
| Rectal therapy only | 1 (1) | 1 (2) | 0 (0) | 0 (0) |
| Multiple Therapies | 34 (22) | 8 (13) | 24 (40) | 2 (6) |
| Biologic + IM | 23 (14) | 2 (3) | 18 (30) | 2 (6) |
| Corticosteroid + Biologic/IM | 6 (4) | 4 (6) | 2 (3) | 0 (0) |
| Corticosteroid + 5-ASA | 2 (1) | 1 (1) | 1 (2) | 0 (0) |
| Corticosteroid + 5-ASA + IM | 1 (1) | 0 (0) | 1 (1) | 0 (0) |
| 5-ASA + IM | 2 (1) | 1 (1) | 2 (3) | 0 (0) |
| **Median CRP** | | | | |
| Total [Q1-Q3] | 5.1 [1.8 - 13.0] | 6.8 [1.3 - 17.0] | 4.2 [1.4 - 11.0] | 4.5 [3.3 - 8.5] |
| Crohn’s disease [Q1-Q3] | 4.3 [1.7 – 12.2] | 6.7 [1.1 – 13.7] | 4.3 [1.8 – 12.4] | 4.0 [3.0 – 6.5] |
| Ulcerative colitis [Q1-Q3] | 8.0 [3.9 - 13.5] | 6.0 [ 4.4 – 14.1] | 5.1 [0.8 -9.4] | 0.0 [0.0 -0.0] |
| Not reported (*n*, % of total) | 42 (27) | 3 (5) | 24 (40) | 20 (57) |

*Immunomodulators include thiopurine or methotrexate

^#^ Includes biosimilar CPT-13, originator infliximab, adalimumab, golimumab

^+^Clinical trial drugs included rizankizumab and upacitanib

Abbreviations: IM immunomodulator; TNF tumor necrosis factor; CRP C-reactive protein.

**Supplemental Table 2. Disease activity measures, resource utilization, change in management and surgical consultations per recruitment site**

| **Outcome measures** | **Combined Population**  ***n* = 158 (100%)** | | **Calgary (Canada)**  ***n* = 63 (40%)** | | **Melbourne and Adelaide (Australia)**  ***n* = 60 (38%)** | | **Tel Aviv (Israel)**  ***n* = 35 (22%)** | |
| --- | --- | --- | --- | --- | --- | --- | --- | --- |
|  | **US* only**  ***n* = 128** | **US + SIG**  ***n* = 30** | **US only**  ***n* = 39** | **US + SIG**  ***n* = 24** | **US only**  ***n* = 54** | **US + SIG**  ***n* = 6** | **US only**  ***n* = 35** | **US + SIG**  ***n* = 0** |
| **Disease Activity Measure** | | | | | | | | |
| **Active inflammation**  (*n*, %) | 82 (64) | 20 (67) | 25 (64) | 17 (71) | 28 (52) | 3 (50) | 29 (83) | 0 (0) |
| **Median Maximal BWT**  (mm, [Q1-Q3]) | 4.2 [2.8-5.8] | 4.9 [3.0-7.2] | 6.0 [3.1-7.9] | 5.7 [3.3-6.4] | 3.5 [2.5-5.1] | 2.9 [2.2-3.3] | 3.5 [2.8-5.0] | 0 [0.0-0.0] |
| **Presence of stricture** (*n*, %) | 21 (16) | 1 (3) | 9 (23) | 1 (4) | 10 (19) | 0 (0) | 2 (6) | 0 (0) |
| **Resource utilization** | | | | | | | | |
| **Avoided urgent endoscopy**  (*n*, %) | 110 (86) | 21 (70) | 32 (82) | 21 (88) | 49 (91) | 0 (0) | 29 (83) | 0 (0) |
| **Further investigations^+^**  (*n*, %) | 84 (66) | 20 (67) | 25 (64) | 18 (75) | 42 (78) | 2 (33) | 17 (49) | 0 (0) |
| **Required non-urgent endoscopy**  (*n*, %) | 35 (27) | 24 (80) | 10 (26) | 19 (79) | 25 (46) | 5 (83) | 0 (0) | 0 (0) |
| **Change in medication** | | | | | | | | |
| **Corticosteroid start/continued**  (*n*, %) | 13 (10) | 1 (3) | 7 (18) | 0 (0) | 5 (9) | 1 (17) | 1 (3) | 0 (0) |
| **Immunosuppression start/optimization**  (*n*, %) | 31 (24) | 3 (10) | 3 (8) | 2 (8) | 26 (48) | 1 (17) | 2 (6) | 0 (0) |
| **Biologic start/optimization**  (*n*, %) | 64 (50) | 3 (10) | 19 (49) | 3 (13) | 30 (56) | 0 (0) | 15 (43) | 0 (0) |
| **JAK inhibitor start/optimization**  (*n*, %) | 0 | 3 (10) | 0 (0) | 3 (13) | 0 (0) | 0 (0) | 0 (0) | 0 (0) |
| **De-escalation of therapy** (*n*, %) | 5 (4) | 0 (0) | 2 (5) | 0 (0) | 3 (6) | 0 (0) | 0 (0) | 0 (0) |
| **Surgical consultation**  (*n*, %) | 4 (3) | 0 (0) | 4 (14) | 0 (0) | 0 (0) | 0 (0) | 0 (0) | 0 (0) |

Abbreviations: BWT bowel wall thickness
